# (4-Hy­droxy-3,5-di­methyl­phen­yl)(phen­yl)methanone

**DOI:** 10.1107/S1600536813028444

**Published:** 2013-10-19

**Authors:** C. S. Dileep, T. Prashanth, S. Jeyaseelan, S. A. Khanum, M. A. Sridhar

**Affiliations:** a Department of Studies in Physics, Manasagangotri, University of Mysore, Mysore 570 006, India; b Department of Chemistry, Yuvaraja’s College, University of Mysore, Mysore 570 005, India; c Department of Physics, St Philomena’s College, Mysore, India

## Abstract

In the mol­ecule of the title compound, C_15_H_14_O_2_, the dihedral angle between the benzene and phenyl rings is 61.27 (8)°. In the crystal, O—H⋯O and weak C—H⋯O hydrogen bonds link the mol­ecules into chains extending along the *c*-axis direction.

## Related literature

For the biological activity of benzo­phenone derivatives, see: Naldoni *et al.* (2009 ▶); Naveen *et al.* (2006 ▶); Selvi *et al.* (2003 ▶). For bond-length and angle data in a related structure, see: Mahendra *et al.* (2005 ▶).
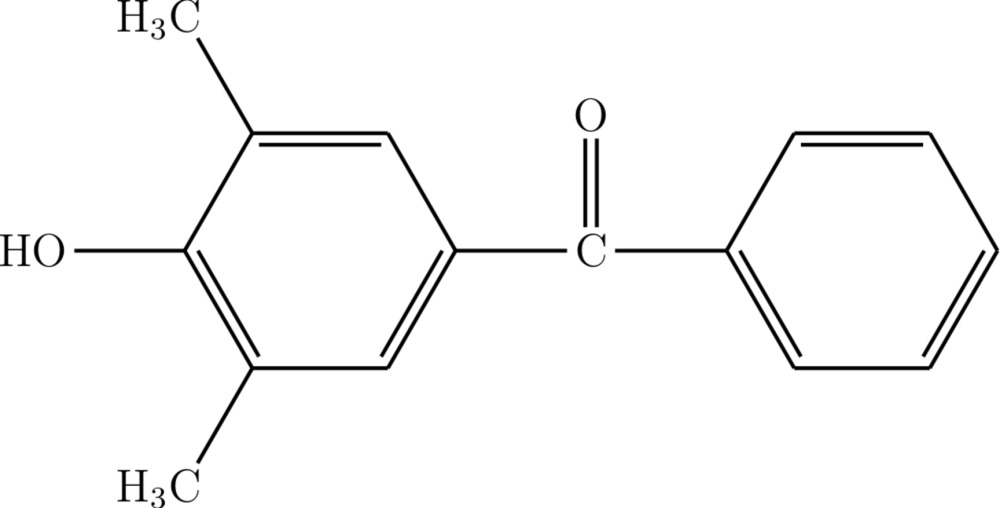


## Experimental

### 

#### Crystal data

C_15_H_14_O_2_
*M* *_r_* = 226.26
Monoclinic, 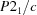

*a* = 4.7741 (13) Å
*b* = 15.198 (4) Å
*c* = 17.274 (5) Å
β = 95.275 (12)°
*V* = 1248.0 (6) Å^3^
*Z* = 4
Mo *K*α radiation
μ = 0.08 mm^−1^
*T* = 293 K
0.30 × 0.25 × 0.20 mm

#### Data collection

Bruker APEXII CCD area-detector diffractometer
2238 measured reflections
2238 independent reflections
1777 reflections with *I* > 2σ(*I*)

#### Refinement

*R*[*F* ^2^ > 2σ(*F* ^2^)] = 0.041
*wR*(*F* ^2^) = 0.140
*S* = 1.02
2238 reflections
157 parameters
H-atom parameters constrained
Δρ_max_ = 0.15 e Å^−3^
Δρ_min_ = −0.13 e Å^−3^

### 

Data collection: *APEX2* (Bruker, 2009 ▶); cell refinement: *SAINT* (Bruker, 2009 ▶); data reduction: *SAINT*; program(s) used to solve structure: *SHELXS97* (Sheldrick, 2008 ▶); program(s) used to refine structure: *SHELXL97* (Sheldrick, 2008 ▶); molecular graphics: *PLATON* (Spek, 2009 ▶); software used to prepare material for publication: *SHELXL97*.

## Supplementary Material

Crystal structure: contains datablock(s) global, I. DOI: 10.1107/S1600536813028444/zs2279sup1.cif

Structure factors: contains datablock(s) I. DOI: 10.1107/S1600536813028444/zs2279Isup2.hkl

Supplementary material file. DOI: 10.1107/S1600536813028444/zs2279Isup3.cml

Additional supplementary materials:  crystallographic information; 3D view; checkCIF report

## Figures and Tables

**Table 1:** Hydrogen-bond geometry (Å, °)

*D*—H⋯*A*	*D*—H	H⋯*A*	*D*⋯*A*	*D*—H⋯*A*
O5—H5⋯O11^i^	0.82	2.03	2.7528 (17)	147
C13—H13⋯O5^ii^	0.93	2.55	3.301 (2)	138

Symmetry codes: (i) 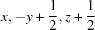; (ii) 
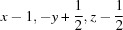
.

**Fig. 1.**
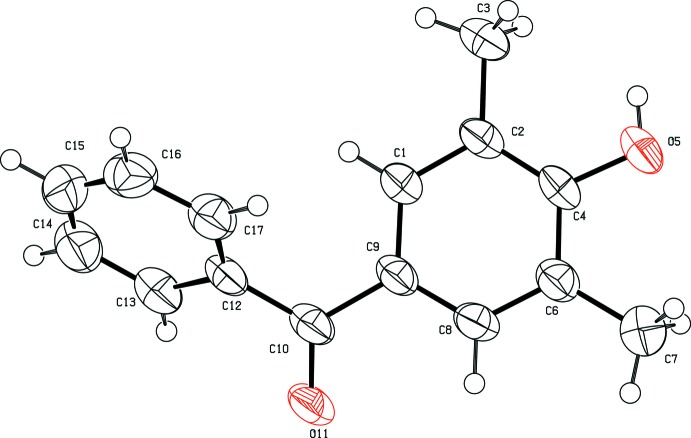
Molecular conformation and atom numbering scheme for the title compound, showing 50% probability displacement ellipsoids.

**Fig. 2.**
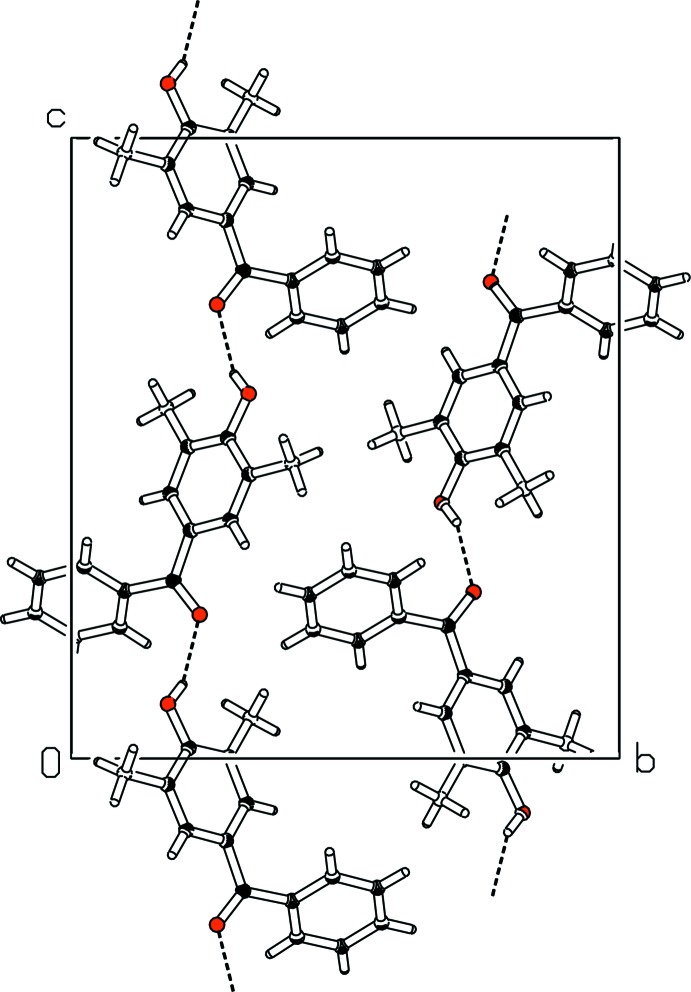
Packing diagram of the molecule in the unit cell viewed down the a axis.

## supplementary crystallographic information

### 1. Comment

Benzophenone and its derivatives show various biological activities such as anti-fungal and anti-inflamatory (Naldoni *et al.*, 2009 and Selvi *et al.*, 2003). The presence of various substituents in the benzophenone nucleus is essential in determining the quantitative structure-activity relationships for these systems. The competence of benzophenones as chemotherapeutic agents, especially as inhibitors of HIV-1 reverse transcriptase RT, cancer and inflammation, is well established and their chemistry has been studied extensively. In addition, methyl-substituted benzophenones exhibit chemotherapeutical activity against fungi. Some studies were carried out to show that these compounds exhibit anti-fungal properties (Naveen *et al.*, 2006) In view of its extensive background, the title compound, C_15_H_14_O_2_, was prepared and characterized by single-crystal X-ray diffraction and the structure is reported herein.

In the molecular structure of this compound (Fig. 1), bond lengths and angles do not show large deviations from and are comparable with those reported for a similar structure (Mahendra *et al.*, 2005). The dihedral angle between the two benzene rings is 61.27 (8)°. The crystal structure is stabilized by intermolecular O—H···O and weak C—H···O hydrogen bonds, forming one-dimensional chains extending along the *c* axis in the unit cell (Fig. 2).

### 2. Experimental

(4-Hydroxy-3,5-dimethyl-phenyl)phenyl-methanone was synthesized by the Fries rearrangement. 2,6-Dimethylphenyl benzoate (0.022 mol) was mixed with anhydrous aluminium chloride (0.044 mol) and fused at 150–170 °C under dry conditions for about 2–3 h. The reaction mixture was then cooled to room temperature and quenched with 6 M HCl in the presence of ice water. The reaction mixture was stirred for about 2–3 h, then filtered and the product was recrystallized from ethanol to obtain colourless crystals.

### 3. Refinement

All H-atoms were located from difference maps but were then positioned geometrically and refined using a riding model, with C—H = 0.93–0.96 Å and and O—H = 0.82 Å, with *U*_iso_(H) = 1.2*U*_eq_(C) (aromatic) or 1.5*U*_eq_(C) (methyl or O). One reflection (0 2 0) was considered to be seriously affected by beamstop interference and was omitted from the data set.

### Crystal data

**Table:**

C_15_H_14_O_2_	*F*(000) = 480
*M**_r_* = 226.26	*D*_x_ = 1.204 Mg m^−^^3^
Monoclinic, *P*2_1_/*c*	Mo *K*α radiation, λ = 0.71073 Å
Hall symbol: -P 2ybc	Cell parameters from 2238 reflections
*a* = 4.7741 (13) Å	θ = 1.8–25.3°
*b* = 15.198 (4) Å	µ = 0.08 mm^−^^1^
*c* = 17.274 (5) Å	*T* = 293 K
β = 95.275 (12)°	Block, colorless
*V* = 1248.0 (6) Å^3^	0.30 × 0.25 × 0.20 mm
*Z* = 4	

### Data collection

**Table:**

Bruker APEXII CCD area-detector diffractometer	1777 reflections with *I* > 2σ(*I*)
Radiation source: fine focus sealed tube	*R*_int_ = 0.000
Graphite monochromator	θ_max_ = 25.3°, θ_min_ = 1.8°
ω and φ scans	*h* = −5→5
2238 measured reflections	*k* = 0→18
2238 independent reflections	*l* = 0→20

### Refinement

**Table:**

Refinement on *F*^2^	Secondary atom site location: difference Fourier map
Least-squares matrix: full	Hydrogen site location: inferred from neighbouring sites
*R*[*F*^2^ > 2σ(*F*^2^)] = 0.041	H-atom parameters constrained
*wR*(*F*^2^) = 0.140	*w* = 1/[σ^2^(*F*_o_^2^) + (0.0867*P*)^2^ + 0.1512*P*] where *P* = (*F*_o_^2^ + 2*F*_c_^2^)/3
*S* = 1.02	(Δ/σ)_max_ = 0.011
2238 reflections	Δρ_max_ = 0.15 e Å^−^^3^
157 parameters	Δρ_min_ = −0.13 e Å^−^^3^
0 restraints	Extinction correction: *SHELXL97* (Sheldrick, 2008), FC^*^=KFC[1+0.001XFC^2^Λ^3^/SIN(2Θ)]^-1/4^
Primary atom site location: structure-invariant direct methods	Extinction coefficient: 0.020 (6)

### Special details

**Table:**

Geometry. Bond distances, angles etc. have been calculated using the rounded fractional coordinates. All su's are estimated from the variances of the (full) variance-covariance matrix. The cell esds are taken into account in the estimation of distances, angles and torsion angles
Refinement. Refinement on F^2^ for ALL reflections except those flagged by the user for potential systematic errors. Weighted R-factors wR and all goodnesses of fit S are based on F^2^, conventional R-factors R are based on F, with F set to zero for negative F^2^. The observed criterion of F^2^ > 2sigma(F^2^) is used only for calculating -R-factor-obs etc. and is not relevant to the choice of reflections for refinement. R-factors based on F^2^ are statistically about twice as large as those based on F, and R-factors based on ALL data will be even larger.

### Fractional atomic coordinates and isotropic or equivalent isotropic displacement parameters (Å^2^)

**Table:**

	*x*	*y*	*z*	*U*_iso_*/*U*_eq_	
O5	0.0022 (3)	0.17634 (8)	0.08784 (6)	0.0634 (4)	
O11	−0.1206 (3)	0.26574 (7)	−0.26812 (6)	0.0637 (5)	
C1	−0.2554 (3)	0.31934 (9)	−0.07292 (8)	0.0450 (5)	
C2	−0.2212 (3)	0.28659 (10)	0.00211 (8)	0.0454 (5)	
C3	−0.3756 (4)	0.32668 (13)	0.06525 (9)	0.0641 (6)	
C4	−0.0447 (3)	0.21413 (10)	0.01672 (8)	0.0454 (5)	
C6	0.0962 (3)	0.17493 (10)	−0.04227 (8)	0.0466 (5)	
C7	0.2852 (4)	0.09705 (12)	−0.02409 (11)	0.0663 (7)	
C8	0.0532 (3)	0.20949 (10)	−0.11568 (8)	0.0463 (5)	
C9	−0.1201 (3)	0.28197 (9)	−0.13271 (8)	0.0430 (5)	
C10	−0.1712 (3)	0.31344 (9)	−0.21370 (8)	0.0458 (5)	
C12	−0.2919 (3)	0.40255 (9)	−0.22965 (8)	0.0440 (5)	
C13	−0.5127 (4)	0.41181 (11)	−0.28726 (9)	0.0551 (6)	
C14	−0.6304 (4)	0.49312 (14)	−0.30321 (11)	0.0692 (7)	
C15	−0.5237 (5)	0.56614 (12)	−0.26413 (11)	0.0684 (7)	
C16	−0.3009 (4)	0.55860 (11)	−0.20861 (11)	0.0634 (6)	
C17	−0.1870 (4)	0.47666 (10)	−0.19002 (9)	0.0535 (5)	
H1	−0.37210	0.36770	−0.08360	0.0540*	
H3A	−0.49780	0.37240	0.04370	0.0960*	
H3B	−0.24260	0.35110	0.10450	0.0960*	
H3C	−0.48500	0.28220	0.08800	0.0960*	
H5	−0.08640	0.20290	0.11900	0.0950*	
H7A	0.17730	0.04930	−0.00590	0.0990*	
H7B	0.43120	0.11290	0.01540	0.0990*	
H7C	0.36800	0.07920	−0.07020	0.0990*	
H8	0.14290	0.18370	−0.15550	0.0560*	
H13	−0.58120	0.36280	−0.31520	0.0660*	
H14	−0.78260	0.49860	−0.34060	0.0830*	
H15	−0.60310	0.62110	−0.27540	0.0820*	
H16	−0.22610	0.60850	−0.18340	0.0760*	
H17	−0.04030	0.47130	−0.15100	0.0640*	

### Atomic displacement parameters (Å^2^)

**Table:**

	*U*^11^	*U*^22^	*U*^33^	*U*^12^	*U*^13^	*U*^23^
O5	0.0797 (8)	0.0818 (8)	0.0287 (6)	0.0183 (6)	0.0057 (5)	0.0122 (5)
O11	0.1119 (10)	0.0537 (7)	0.0258 (6)	0.0055 (6)	0.0085 (6)	−0.0042 (5)
C1	0.0549 (9)	0.0495 (8)	0.0305 (8)	0.0022 (6)	0.0031 (6)	0.0003 (6)
C2	0.0523 (9)	0.0571 (9)	0.0270 (8)	−0.0006 (7)	0.0049 (6)	−0.0013 (6)
C3	0.0787 (12)	0.0819 (12)	0.0333 (9)	0.0148 (9)	0.0137 (8)	0.0005 (8)
C4	0.0524 (9)	0.0586 (9)	0.0246 (7)	−0.0031 (7)	−0.0004 (6)	0.0037 (6)
C6	0.0520 (9)	0.0545 (8)	0.0330 (8)	0.0007 (6)	0.0016 (6)	0.0001 (6)
C7	0.0764 (12)	0.0698 (11)	0.0525 (11)	0.0179 (9)	0.0048 (9)	0.0067 (8)
C8	0.0570 (9)	0.0526 (8)	0.0302 (8)	0.0004 (7)	0.0091 (7)	−0.0040 (6)
C9	0.0565 (9)	0.0474 (8)	0.0248 (7)	−0.0036 (6)	0.0027 (6)	−0.0009 (6)
C10	0.0627 (10)	0.0481 (8)	0.0269 (8)	−0.0086 (7)	0.0057 (6)	−0.0029 (6)
C12	0.0586 (9)	0.0497 (8)	0.0244 (7)	−0.0060 (6)	0.0078 (6)	0.0018 (6)
C13	0.0649 (10)	0.0651 (10)	0.0351 (9)	−0.0031 (8)	0.0036 (8)	0.0004 (7)
C14	0.0708 (12)	0.0884 (14)	0.0481 (10)	0.0165 (10)	0.0036 (9)	0.0103 (9)
C15	0.0848 (13)	0.0628 (11)	0.0610 (12)	0.0199 (9)	0.0259 (10)	0.0163 (9)
C16	0.0850 (13)	0.0479 (9)	0.0605 (11)	−0.0057 (8)	0.0247 (10)	−0.0041 (8)
C17	0.0649 (10)	0.0534 (9)	0.0423 (9)	−0.0055 (7)	0.0050 (7)	−0.0025 (7)

### Geometric parameters (Å, º)

**Table:**

O5—C4	1.3560 (18)	C14—C15	1.372 (3)
O11—C10	1.2286 (18)	C15—C16	1.370 (3)
O5—H5	0.8200	C16—C17	1.385 (2)
C1—C2	1.384 (2)	C1—H1	0.9300
C1—C9	1.389 (2)	C3—H3A	0.9600
C2—C4	1.396 (2)	C3—H3B	0.9600
C2—C3	1.501 (2)	C3—H3C	0.9600
C4—C6	1.404 (2)	C7—H7A	0.9600
C6—C7	1.504 (2)	C7—H7B	0.9600
C6—C8	1.371 (2)	C7—H7C	0.9600
C8—C9	1.393 (2)	C8—H8	0.9300
C9—C10	1.477 (2)	C13—H13	0.9300
C10—C12	1.488 (2)	C14—H14	0.9300
C12—C17	1.387 (2)	C15—H15	0.9300
C12—C13	1.389 (2)	C16—H16	0.9300
C13—C14	1.375 (3)	C17—H17	0.9300
			
C4—O5—H5	109.00	C2—C1—H1	119.00
C2—C1—C9	121.76 (13)	C9—C1—H1	119.00
C1—C2—C3	120.74 (14)	C2—C3—H3A	109.00
C1—C2—C4	118.07 (13)	C2—C3—H3B	109.00
C3—C2—C4	121.18 (13)	C2—C3—H3C	109.00
O5—C4—C6	115.31 (13)	H3A—C3—H3B	109.00
C2—C4—C6	121.68 (13)	H3A—C3—H3C	110.00
O5—C4—C2	123.01 (13)	H3B—C3—H3C	109.00
C4—C6—C8	117.90 (14)	C6—C7—H7A	110.00
C7—C6—C8	122.06 (14)	C6—C7—H7B	109.00
C4—C6—C7	120.04 (13)	C6—C7—H7C	110.00
C6—C8—C9	122.28 (13)	H7A—C7—H7B	109.00
C1—C9—C10	121.61 (13)	H7A—C7—H7C	109.00
C8—C9—C10	119.95 (12)	H7B—C7—H7C	109.00
C1—C9—C8	118.31 (13)	C6—C8—H8	119.00
O11—C10—C9	120.44 (13)	C9—C8—H8	119.00
C9—C10—C12	119.84 (12)	C12—C13—H13	120.00
O11—C10—C12	119.71 (13)	C14—C13—H13	120.00
C10—C12—C13	118.72 (13)	C13—C14—H14	120.00
C13—C12—C17	119.15 (14)	C15—C14—H14	120.00
C10—C12—C17	122.11 (13)	C14—C15—H15	120.00
C12—C13—C14	120.27 (16)	C16—C15—H15	120.00
C13—C14—C15	120.12 (18)	C15—C16—H16	120.00
C14—C15—C16	120.39 (18)	C17—C16—H16	120.00
C15—C16—C17	120.04 (16)	C12—C17—H17	120.00
C12—C17—C16	119.96 (16)	C16—C17—H17	120.00
			
C9—C1—C2—C3	−178.40 (15)	C1—C9—C10—O11	−158.32 (15)
C9—C1—C2—C4	0.1 (2)	C1—C9—C10—C12	20.0 (2)
C2—C1—C9—C8	0.2 (2)	C8—C9—C10—O11	17.4 (2)
C2—C1—C9—C10	176.00 (13)	C8—C9—C10—C12	−164.22 (13)
C1—C2—C4—O5	−179.48 (14)	O11—C10—C12—C13	45.2 (2)
C1—C2—C4—C6	0.2 (2)	O11—C10—C12—C17	−133.42 (17)
C3—C2—C4—O5	−1.0 (2)	C9—C10—C12—C13	−133.19 (15)
C3—C2—C4—C6	178.64 (15)	C9—C10—C12—C17	48.2 (2)
O5—C4—C6—C7	−0.5 (2)	C10—C12—C13—C14	179.61 (15)
O5—C4—C6—C8	178.98 (14)	C17—C12—C13—C14	−1.8 (2)
C2—C4—C6—C7	179.84 (15)	C10—C12—C17—C16	178.02 (15)
C2—C4—C6—C8	−0.7 (2)	C13—C12—C17—C16	−0.6 (2)
C4—C6—C8—C9	1.0 (2)	C12—C13—C14—C15	2.3 (3)
C7—C6—C8—C9	−179.55 (15)	C13—C14—C15—C16	−0.5 (3)
C6—C8—C9—C1	−0.7 (2)	C14—C15—C16—C17	−1.8 (3)
C6—C8—C9—C10	−176.64 (14)	C15—C16—C17—C12	2.4 (3)

### Hydrogen-bond geometry (Å, º)

**Table:**

*D*—H···*A*	*D*—H	H···*A*	*D*···*A*	*D*—H···*A*
O5—H5···O11^i^	0.82	2.03	2.7528 (17)	147
C13—H13···O5^ii^	0.93	2.55	3.301 (2)	138

Symmetry codes: (i) *x*, −*y*+1/2, *z*+1/2; (ii) *x*−1, −*y*+1/2, *z*−1/2.
